# Quality of life and depression of people living with type 2 diabetes mellitus and those at low and high risk for type 2 diabetes: findings from the Study to Help Improve Early evaluation and management of risk factors Leading to Diabetes (SHIELD)

**DOI:** 10.1111/j.1742-1241.2008.01703.x

**Published:** 2008-04

**Authors:** S Grandy, R H Chapman, K M Fox

**Affiliations:** 1 AstraZeneca LP Wilmington, DE, USA; 2 IMS Health – US HEOR (formerly ValueMedics Research) Falls Church, VA, USA; 3 Strategic Healthcare Solutions, LLC Monkton, MD, USA

## Abstract

**Objectives:**

This study compared health-related quality of life (HRQoL) and depression among individuals with type 2 diabetes mellitus (T2D) and those at low or high risk for T2D.

**Methods:**

Respondents in a population-based US 2004 survey reported whether they had T2D (*n* = 3530) or risk factors for T2D [abdominal obesity, body mass index (BMI) ≥ 28 kg/m^2^, dyslipidaemia, hypertension and history of cardiovascular disease]. Respondents without T2D were stratified into low risk (0–2 risk factors, *n* = 5335) and high risk (3–5 risk factors, *n* = 5051). SF-12 version 2 (SF-12) and Patient Health Questionnaire (PHQ)-9 were used to measure HRQoL and depression. Mean scores were compared across the three groups using analysis of variance. Linear regression identified factors associated with SF-12 Physical and Mental Component Summary scores (PCS and MCS), adjusting for age, gender, race, income, geographic region, household size, BMI and group.

**Results:**

Respondents were mostly women (60%) with mean age of 54 years. Mean PCS scores for T2D and high risk (39.5 and 41.7, respectively) were significantly lower than for low risk (50.6, p < 0.001). After adjustment, high-risk and T2D groups were associated with lower PCS and MCS scores compared with low risk group (p < 0.05). Mean PHQ-9 scores and per cent with moderate-to-severe depression were significantly higher for T2D and high risk than for low risk (p < 0.01).

**Conclusions:**

Health-related quality of life and depression scores in T2D were similar to those at high risk, and indicated significant decrements in physical health and greater depression compared with low-risk respondents.

**Disclosures:**

SHIELD, the SHIELD Study Group and the preparation of this manuscript were supported by funding from AstraZeneca LP. Dr Grandy is an employee of AstraZeneca LP and Drs Chapman and Fox are independent consultants who received research funds to conduct this analysis.

**What's known**

Previous studies have documented that diabetes, its treatment, complications and comorbidities adversely affect health-related quality of life among patients with diabetes. Additionally, depressive symptoms are common among patients with diabetes.

**What's new**

This study provides new evidence of the burden of being at high risk for diabetes on health-related quality of life and depressive symptoms, bringing attention and recognition that quality of life is affected in those not diagnosed with type 2 diabetes but at high risk of developing the disease. Also, the present study observed the impact of type 2 diabetes on quality of life in a large representative sample of individuals treated in usual clinical practice.

## Introduction

Diabetes mellitus affects approximately 20.6 million people in the USA (1). Of these, an estimated 6.2 million, nearly one-third of the affected population, are unaware that they have the disease. Diabetes mellitus is the fifth deadliest disease in the USA, and more than a million people develop the disease each year. Economic costs associated with diabetes mellitus are estimated to be approximately $132 billion annually (2).

The primary risk factors for the development of type 2 diabetes mellitus (T2D) are overweight, sedentary, ≥ age of 45 years and/or a family history of diabetes mellitus (1). African Americans, Latinos and Native Americans are at increased risk, as are women who have had babies weighing more than 9 pounds at birth (1). Diabetes mellitus is a chronic disease that requires ongoing monitoring and treatment. Its associated complications include blindness, kidney disease, nerve damage and cardiovascular disease (CVD). CVD and stroke account for 65% of deaths in persons with diabetes mellitus, and individuals with diabetes mellitus experience a CVD death rate 2–4 times higher than adults without diabetes mellitus (3).

As increasingly evidenced in the literature, diabetes mellitus substantially affects patients’ health-related quality of life (HRQoL) (4–7). More than two dozen different quality of life instruments have been used to report the HRQoL of patients with diabetes mellitus. For those living with diabetes mellitus, the impact of treatments, complications and comorbidities has been documented to adversely affect HRQoL (8). Additionally, depressive symptoms are common among patients with diabetes (9). However, limited information is available on the HRQoL and depression of those without T2D but at high risk for developing this condition. The Study to Help Improve Early evaluation and management of risk factors Leading to Diabetes (SHIELD) is a 5-year, survey-based study conducted to better understand patterns of health behaviour, knowledge and attitudes of people living with diabetes mellitus, and those with varying levels of cardiometabolic risk. The present investigation is a cross-sectional analysis designed to assess and compare the HRQoL and depression among individuals with T2D and those at low or high risk for T2D to determine if the burden is similar among at-risk individuals. Findings from this study will provide a better understanding of the unmet medical needs and burden of illness in the T2D and at-risk populations.

## Methods

A detailed questionnaire (baseline survey) was mailed in 2004 to 22,001 individuals, age 18 years and older who were identified with diabetes or risk factors associated with diabetes. The baseline survey assessed comorbid conditions, health status, knowledge, attitudes and current behaviours related to general health and diabetes, and HRQoL and depression. A response rate of 80% was achieved for the baseline survey. A detailed description of the SHIELD methodology has been published elsewhere (10,11).

The sample source for the baseline survey was selected from respondents to a screening survey mailed to a stratified random sample of 200,000 US households, representative of the US population for age of head of household, income, household size, urban density and census region, identified by the Taylor Nelson Sofres National Family Opinion panel (TNS, Greenwich, CT). The screening questionnaire was completed by the head of household, who answered for up to four adult household members. Responses were received from 211,097 adults for a response rate of 64%. Postweighting of the SHIELD data was performed to correct for over- or under-sampling of some demographic groups and to ensure that respondents represented the US Census population (12) in terms of geographic residence, age of the head of household, household size and income.

Respondents to the screening and baseline surveys were classified according to diagnosis of diabetes (type 1 or type 2) and risk factors associated with increased risk of T2D. Recognised risk factors, derived from the literature, national guidelines and expert opinion (13,14), included: (i) abdominal obesity (defined as waist circumference ≥ 97 cm for men and ≥ 89 cm for women), (ii) body mass index (BMI) ≥ 28 kg/m^2^ (general obesity), (iii) diagnosis of dyslipidaemia (cholesterol problems), (iv) diagnosis of hypertension (high blood pressure) and (v) diagnosis of CVD (defined as one or more of heart disease, myocardial infarction, narrow or blocked arteries, stroke, coronary artery bypass graft surgery, angioplasty, stents or surgery to clear arteries). Receiver operating characteristic (ROC) curves were computed for all possible cutoff points for BMI and waist circumference and the level for which the area under the curve was maximised (maximises the number of T2D respondents correctly classified) was chosen as the optimal cutoff point. The abdominal obesity and BMI cutoff-points are similar to those recommended by the National Cholesterol Education Program and National Heart, Lung, and Blood Institute (14,15). Stepwise logistic regression analyses verified that these five risk factors were independently and equally associated with diabetes diagnosis in the SHIELD population. Respondents with zero, one or two of the five risk factors were further classified as low risk for diabetes and respondents with 3–5 risk factors were classified as high risk. ROC curves for all possible cutoff points of the risk factor score were evaluated and maximised when three or more risk factors were present.

### Measures

#### 12-item Short Form version 2

The SF-12v2™ Health Survey (SF-12; Quality Metric Inc., Lincoln, RI, USA), the short form of the widely used SF-36, is a brief and reliable measure of overall health status (16). The SF-12 measures eight domains of health: physical functioning, role limitations because of physical health (role-physical), bodily pain, general health perceptions, vitality, social functioning, role limitations because of emotional problems (role-emotional) and mental health. The recall period for the SF-12 was the past 4 weeks. SF-12 responses were scored from 0 to 100 on each of the domains, as well as for the Physical Component Summary (PCS) scale and Mental Component Summary (MCS) scale. Higher scores indicate better HRQoL. To simplify comparisons with the general population, norm-based scoring was used. In norm-based scoring, scores are linearly transformed to a scale with a mean of 50 and standard deviation (SD) of 10 for the general US population (16).

#### Patient Health Questionnaire

The Patient Health Questionnaire (PHQ)-9 focuses on the nine signs and symptoms of depression from the Diagnostic and Statistical Manual of Mental Disorders, Fourth edition (DSM-IV) (17). The PHQ-9 is a dual-purpose instrument that is used to establish a provisional depressive disorder diagnosis as well as provide a symptom severity score. Higher scores indicate increasing severity of depression. For a diagnosis of depression, five or more items must be scored as present more than half of the days or nearly every day. PHQ-9 scores of 5–9 indicate minimal depressive symptoms, scores of 10–14 indicate minor depression or major depression that is mild, scores of 15–19 is major depression, moderately severe and scores ≥ 20 indicate major depression, severe (17).

### Statistical analyses

For each group (T2D, high risk, low risk), we reported mean scores overall for SF-12 PCS and MCS and PHQ-9. Statistical comparisons across groups were made using analysis of variance (ANOVA) with Fisher's least significant difference *post hoc* testing, with p < 0.01 considered significant. Significance testing comparing groups on HRQoL and depression scores was performed, with emphasis on comparisons between the T2D group and the high- and low-risk groups. Additionally, multivariable linear regression modelling was used to identify those factors that most affected respondents’ HRQoL. Dependent HRQoL variables were the PCS score and the MCS score of the SF-12. Explanatory factors included socio-demographic factors such as age, gender, race, geographic region, household income, as well as BMI category (normal weight, overweight or obese) and group status (low risk, high risk or T2D).

## Results

Greater than 70% of each cohort (5335/7403 for low risk, 5051/6742 for high risk and 3530/5000 for T2D) completed the SF-12 and PHQ-9 questions. T2D respondents were significantly older than high- and low-risk respondents (Table 1). The T2D respondents also were significantly less likely to be white, have some college or higher education, and more likely to have lower incomes than high- and low-risk respondents. There was a significantly greater proportion of women in the low-risk group compared with T2D and high-risk groups (Table 1). A history of cardiovascular events had the lowest prevalence among all groups, while abdominal obesity and BMI obesity were concomitant in the majority of respondents in each group (Table 1).

**Table 1:** Patient characteristics of SHIELD respondents completing the SF-12 and PHQ-9

Characteristics	T2D (*n* = 3530)	High risk (*n* = 5051)	Low risk (*n* = 5335)
Age, years, mean (SD)	59.7 (13.0)	58.5 (14.6)**	46.2 (16.0)*
Gender, % women	57	56	65*
Race, % white	85	89**	88*
Education, % with some college or higher	65	68**	75*
Income, % with < $40,000/year	51	46**	36*
**Risk factors**			
Abdominal obesity, %	86	96	46*
BMI ≥ 28 kg/m^2^, %	77	88	34*
Hypertension, %	67	76	12*
Dyslipidaemia, %	73	81	20*
Cardiac event, %	29	36	4*

* p < 0.01, T2D vs. low risk.

** p < 0.01, T2D vs. high risk. SHIELD, Study to Help Improve Early evaluation and management of risk factors Leading to Diabetes; PHQ, Patient Health Questionnaire; T2D, type 2 diabetes mellitus; BMI, body mass index.

### SF-12 PCS and MCS scores

**Figure 1:**
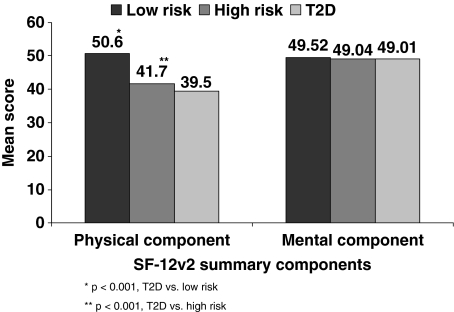
Mean SF-12 Physical Component Summary and Mental Component Summary scores, by group

Mean SF-12 PCS scores for respondents with T2D and those with high risk [39.5 (SD 12.9) and 41.7 (SD 12.3), respectively] were significantly lower than the low-risk group [50.6 (SD 9.9), p < 0.001 for each] (Figure 1). The mean PCS score for T2D respondents was statistically significantly lower than the mean for the high-risk group (p < 0.001).

In contrast, there were no statistically significant differences across groups in mean SF-12 MCS scores (Figure 1). In addition, mean MCS scores were not significantly different from the US population norm of 50, with mean scores of 49.52 (SD = 10.1) for the low-risk group, 49.04 (11.0) for high risk and 49.01 (10.9) for T2D groups.

Examination of SF-12 domain results revealed that scores for the T2D and high-risk groups were significantly lower than the low-risk group scores for all domains (p < 0.01). In addition, mean scores for the T2D group were significantly lower than those for the high-risk group (p < 0.01) in all domains except mental health.

### Linear regression models

Income, age, BMI, gender, race, geographic region, household size and T2D risk status were significantly associated with HRQoL (Table 2). T2D risk status was significantly associated with HRQoL, such that those with T2D had the lowest HRQoL (p < 0.001 vs. low-risk group for PCS and MCS). The decrease in MCS was relatively smaller than in PCS and about equal for the high-risk and T2D groups (approximately −1.7), but significantly different than low-risk group, p < 0.05.

**Table 2:** Multivariate stepwise linear regression analyses of variables impacting health-related quality of life in SHIELD respondents*

	Beta coefficient (SE)	

Significant variables	Physical Component Score†	Mental Component Score†
**Diabetes risk/diagnosis**		
0–2 risk factors	(reference)	(reference)
3–5 risk factors	−4.82 (0.25)	−1.71 (0.24)
Type 2 diabetes	−6.53 (0.27)	−1.72 (0.25)
**Income ($)**		
< 22,500	−7.81 (0.29)	−5.42 (0.28)
22,500–39,999	−4.45 (0.30)	−2.94 (0.28)
40,000–59,999	−2.76 (0.30)	−1.56 (0.29)
60,000–89,999	−1.40 (0.30)	−1.18 (0.29)
≥ 90,000	(reference)	(reference)
**Age (years)**		
18–24	1.80 (0.56)	1.59 (0.53)
25–34	1.00 (0.38)	−1.26 (0.36)
35–44	(reference)	(reference)
45–54	−2.82 (0.30)	1.42 (0.29)
55–64	−4.62 (0.32)	3.62 (0.30)
65–74	−5.27 (0.34)	6.30 (0.32)
≥ 75	−8.78 (0.36)	5.40 (0.35)
**BMI (kg/m**^**2**^**)**		
Underweight (< 18.5)	Dropped	−1.63 (0.81)
Normal weight (18.5–24.9)	(reference)	(reference)
Overweight (25.0–29.9)	Dropped	Dropped
Obese (> 30)	−4.00 (0.21)	−0.75 (0.20)
Female	−2.31 (0.19)	−1.90 (0.18)
Male	(reference)	(reference)
**Race**		
White	(reference)	(reference)
Black	0.95 (0.34)	0.76 (0.33)
Other	−1.34 (0.56)	−1.10 (0.54)
**Geographic region**		
East South Central	−1.41 (0.37)	−0.71 (0.36)
New England	1.01 (0.42)	Dropped
West North Central	Dropped	0.93 (0.34)
East North Central	Dropped	0.52 (0.23)
Pacific	(reference)	(reference)
**Household size**		
1	(reference)	(reference)
3	Dropped	−0.89 (0.24)
≥ 5	−0.71 (0.32)	−1.31 (0.31)

* Scores indicate change from reference group: gender = male, race = white, household income = ≥ $90,000, age = 35–44, BMI = normal weight, group = low risk, geographic region = Pacific, household size = 1.

† p < 0.05 vs. reference group for all values. Dropped = level of variable dropped from model during stepwise regression, no beta coefficient computed. SHIELD, Study to Help Improve Early evaluation and management of risk factors Leading to Diabetes; BMI, body mass index.

For both PCS and MCS, as household incomes decreased, respondents’ HRQoL decreased, such that those with incomes < $22,500 reported the greatest impact on HRQoL (p < 0.001 vs. ≥ $90,000 in both models, Table 2). Increasing age was associated with decreased physical HRQoL, such that individuals age 75 years and older reported the greatest impact on HRQoL (p < 0.001 vs. those aged 35–44), with those aged 18–24 years reporting the highest HRQoL. MCS analysis, however, showed the older age groups, aged 65–74 and ≥ 75 in particular, were more likely to report higher HRQoL in this domain (p < 0.001 for both age groups vs. those aged 35–44, Table 2). For both PCS and MCS, being obese was associated with lower HRQoL, although this decrease was greater for PCS than for MCS. Female gender, other race (Asian, Pacific Islander, American Indian, Aleut Eskimo and Other), East South Central geographic location, and a household size > 5 also were associated with a negative impact on HRQoL.

### PHQ-9 scores

Mean PHQ-9 summed score was significantly lower (indicating lower rates of depression) in the low-risk group (3.66) than in either the high-risk (5.28) or T2D (5.43) groups (p < 0.01, Table 3). When categorised into level of depression, low-risk respondents were more likely to have no-to-minimal depression than the high-risk or T2D groups (69.8% vs. 58.4% and 57.2%, respectively, p < 0.01) and less likely to have moderate-to-severe depression (11.3% vs. 18.1% and 19.6%, respectively, p < 0.01) (Table 3). For each of the nine questions in the PHQ-9, T2D and high-risk respondents reported significantly greater frequency of depressive symptoms than the low-risk respondents, p < 0.01.

**Table 3:** Mean PHQ-9 scores for determining depression diagnosis among SHIELD respondents

PHQ-9 scores	T2D (*n* = 3530)	High risk (*n* = 5051)	Low risk (*n* = 5335)
Summary score, mean (SD)	5.43 (5.72)	5.28 (5.58)	3.86 (4.88)*
% with minimal depression	57.2	58.4	69.8*
% with mild depression	23.2	23.5	18.9
% with moderate-to-severe depression	19.6	18.1	11.3*

* p < 0.01, T2D vs. low risk. PHQ, Patient Health Questionnaire; SHIELD, Study to Help Improve Early evaluation and management of risk factors Leading to Diabetes; T2D, type 2 diabetes mellitus.

## Discussion

In this study, we assessed HRQoL and depression to evaluate the overall burden of T2D and associated risk factors on general health status (SF-12) and more specifically on mental health (PHQ-9). The SHIELD data demonstrate that respondents with T2D and those with a high number of risk factors (3–5) have a self-reported lower HRQoL, compared with those having a lower number of risk factors (0–2), as well as the general population. Respondents with T2D and those with high risk reported significant decrements in physical health HRQoL compared with those with low risk, even after adjusting for modifiable and non-modifiable characteristics. Nearly 50% of those with T2D and those with high risk reported some limitation in the physical component, including work or moderate activities. This study also provided new evidence of decreased HRQoL and increased depressive symptoms among individuals at high risk for T2D but who were not currently diagnosed or treated as well as among those with diagnosed T2D. Ratings of HRQoL and depression in respondents with T2D and those at high risk of T2D were remarkably similar but significantly different from low-risk respondents. This observation may indicate that the accumulation of risk factors for T2D is altering HRQoL before a diagnosis is made.

Additionally, these results showed that health and disease status affected the emotional health of those with T2D and those with high risk in greater proportion than reported by the lower-risk group. In general, HRQoL decrements were greater for physical domains than for emotional or mental domains, but depression as measured by the PHQ-9 was significantly greater in the T2D and high-risk groups. A greater percentage of respondents with T2D and with high risk reported being moderately to severely depressed compared with the low-risk group.

Also evident in this study was the impact that demographic factors, such as age and income, have on individuals’ HRQoL. Those respondents with lower incomes, increased age, and who were obese, at high risk or with a T2D diagnosis reported lower HRQoL scores, after adjusting for non-modifiable risk factors (gender, race, geographic region and household size). These findings confirmed those in the Canadian National Population Survey, (18) where T2D had a greater impact on HRQoL for older ages and low socio-economic status.

The present study confirms the lower HRQoL among T2D respondents that has been observed in other investigations (4–7,18–22). Impaired physical and social functioning but not mental functioning among diabetes mellitus patients was observed in the present study as well as prior studies that utilised the SF-36 general measure (19,20,22). Moreover, the present study observed the impact of T2D on HRQoL in a significantly larger population-based sample (*n* = 3530 T2D) than previous investigations (*n* = 221–254) (19,21). However, these prior investigations typically compared T2D patients with the general population (18–21), whereas this study demonstrated lower HRQoL among T2D and high-risk respondents when compared with low-risk respondents. The greater number of risk factors for T2D significantly impacted HRQoL in our study sample, similar to analyses of the Medical Expenditure Panel Survey, which showed that individuals with a cluster of similar cardiometabolic risk factors had a significant decrease in physical functioning (PCS-12) but not mental functioning (22).

The MCS of the SF-12 did not differentiate risk groups as well as the PCS. The MCS scores were significantly higher for the low-risk than the high-risk or T2D groups, only after adjusting for other covariates such as age, race and gender. Further, the mental health component of the SF-12 did not differentiate risk groups as well as the PHQ-9 depression questionnaire. The PHQ-9 scores demonstrated that the low-risk group had significantly lower scores and smaller per cent of respondents with moderate-to-severe depression compared with the high-risk and T2D groups. Significant differences were observed among the low-risk as compared with the high-risk and T2D respondents for each of the nine items in the PHQ-9. These findings may indicate that the mental health component of the SF-12 may not be sufficiently sensitive to differentiate the groups on the impact of risk status on depression, whereas the PHQ-9 was especially as the MCS is a more general assessment of emotional problems and their impact on work, daily activities and social activities rather than depressive symptoms. The PHQ-9 not only has nine focused questions on depressive symptoms but also is more specific than the MCS for frequency of symptoms (not at all, several days, more than half the days and nearly every day).

This study provides evidence of the impact on HRQoL and depression in a large sample of T2D, high-risk and low-risk groups with a high survey response rate who are representative of the US population. Additionally, the evaluation of HRQoL and depression was carried out using standardised, validated measures so that normative-based results are provided. However, there are limitations to the study that should be considered. Only a small percentage (5–8%) of consumers invited to participate in the TNS panel elect to do so and those who participate are accustomed to completing surveys, leading to the possibility of selection bias. Household panels tend to under-represent the very wealthy and very poor segments of the population, and do not include military or institutionalised individuals. However, these limitations are true for most random sampling and clinically based methodologies. Additionally, the determination of T2D, high-risk and low-risk status was made based upon self-report rather than clinical or laboratory measures for blood glucose, hypertension, cholesterol, height and weight. It should also be noted that other comorbidities in the high-risk respondents, as well as those diagnosed with T2D, may contribute to their overall health burden and HRQoL. However, it is difficult to disentangle the HRQoL effects of such comorbidities from those of the risk factors or T2D, as many of these conditions are related to or a result of having the risk factors or T2D.

## Conclusions

The SHIELD results show that respondents with T2D and those at high risk for T2D report decreased HRQoL and increased depression compared with respondents with lower cardiometabolic risk or the general population, highlighting the unmet need and burden of illness among these groups. It is important to recognise that HRQoL is affected in those not yet diagnosed with T2D but at high risk of developing T2D. There may be potential opportunities to impact the risk factors leading to T2D, which may ameliorate the negative impact on HRQoL and depression and allow interventions to work in these at-risk individuals. Educating clinicians that their high-risk patients who may not yet be diagnosed with T2D are at risk of decreased HRQoL and depression may improve clinicians’ awareness and increase the opportunity for risk management and treatment. It also may be beneficial for clinicians to educate and counsel high-risk patients to manage hypertension and cholesterol levels as well as reduce their weight, which subsequently may lower the patients’ potential for poor HRQoL and depression. Further study is needed to determine the benefit of targeting healthcare interventions in high-risk and T2D patients on reduction of HRQoL impact, and the overall health and economic burden this may ameliorate.
